# ClusterNet: Classifying Single‐Molecule Localization Microscopy Datasets with Graph‐Based Deep Learning of Supracluster Structure

**DOI:** 10.1002/smsc.202500255

**Published:** 2025-10-09

**Authors:** Oliver Umney, Hayley Slaney, Christopher J. M. Williams, Philip Quirke, Michelle Peckham, Alistair P. Curd

**Affiliations:** ^1^ Engineering and Physical Sciences Faculty Services Faculty of Engineering and Physical Sciences University of Leeds Leeds LS2 9JT UK; ^2^ Division of Pathology and Data Analytics Leeds Institute for Medical Research at St James's University of Leeds Leeds LS2 9JT UK; ^3^ Division of Oncology Leeds Institute for Medical Research at St James's University of Leeds Leeds LS9 7TF UK; ^4^ School of Molecular and Cellular Biology Faculty of Biological Sciences University of Leeds Leeds LS2 9JT UK; ^5^ Astbury Centre for Structural and Molecular Biology University of Leeds Leeds LS2 9JT UK

**Keywords:** classification, deep learning, direct stochastic optical reconstruction microscopy, DNA‐PAINT, graph neural network, point cloud, single‐molecule localization microscopy

## Abstract

Single‐molecule localization microscopy (SMLM) data can reveal differences in protein organization between different disease types or samples. Classification of samples is an important task that allows for automated recognition and grouping of data by sample type for downstream analysis. However, methods for classifying structures larger than single clusters of localizations in SMLM point‐cloud datasets are not well developed. A graph‐based deep learning pipeline is presented for classification of SMLM point‐cloud data over a field of view of any size. The pipeline combines features of individual clusters (calculated from their constituent localizations) with the structure formed by the positions of multiple clusters (supracluster structure). This method outperforms previous classification results on a model open‐source DNA‐PAINT dataset, with 99% accuracy. It is also applied to a challenging new SMLM dataset from colorectal cancer tissue. Explainability tools Uniform Manifold Approximation and Projection and SubgraphX allow exploration of the influence of spatial features and structures on classification results, and demonstrate the importance of supracluster structure in classification.

## Graphical abstract

ClusterNet is a graph‐based deep learning pipeline for classifying single‐molecule localization microscopy (SMLM) data. It acts directly on point‐cloud localization data from a field of view of any size, using features of individual clusters of localizations together with their spatial arrangement. It is successfully tested on a model SMLM dataset and on data acquired from biological samples.

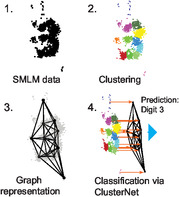

## 1 Introduction

Single‐molecule localization microscopy (SMLM) is widely used for determining protein organization at the nanoscale and has opened up new possibilities for understanding disease and other biological conditions. Unlike conventional imaging techniques, it generates a list of high‐precision coordinates (point cloud) of proteins or other labeled targets in a sample. As SMLM has become more accessible and used for a wider variety of problems, many tools have been developed to analyze these point clouds.^[^ ^1^, ^2^ ^]^

Sample classification is an important step in analysis, allowing for automated recognition of sample type and downstream aggregation and analysis of data from many samples of the same type. Using deep learning (DL) algorithms for this task may also facilitate biological discovery, despite only having sample‐level labels (weakly supervised).^[^ ^3^ ^]^ While DL algorithms have classified SMLM data of complex structures such as whole cells, they have first rendered the data as a pixelated image, sacrificing the full potential of the precision gain of SMLM over conventional imaging, and hence the information available.^[^ ^4^, ^5^ ^]^

Existing pipelines for classifying SMLM point‐cloud datasets do not extend to classification of structures larger than single clusters of localizations. Current methods focus on classifying individual localizations and clusters, using either predetermined (handcrafted) features of clusters, such as cluster area and length,^[^ ^6^, ^7^, ^8^, ^9^, ^10^, ^11^, ^12^, ^13^ ^]^ or more abstract cluster features learnt automatically by a point‐ or graph‐based DL network.^[^ ^14^, ^15^, ^16^ ^]^ However, the arrangement of multiple clusters in a sample (the supracluster structure), as well as the combination of the features of the different clusters, is likely to hold important biological information.

Extending algorithms using handcrafted cluster features to complex localization patterns such as multiple clusters is not straightforward, requiring new calculations for features that would discriminate between them. Point‐ and graph‐based DL pipelines have also been limited to classifying localizations and clusters, and ignore the sample‐level supracluster structure. Particle averaging has been used to classify more complex patterns than handcrafted features have typically described, but is still restricted to classification of single particles with highly consistent structure.^[^ ^17^, ^18^ ^]^ New methods are therefore required for the classification of variable and complex structures in SMLM data, including whole fields of view (FOVs).

Here, we present a DL classification pipeline for SMLM point‐cloud datasets that incorporates supracluster structure. We have developed ClusterNet, a graph‐based DL network, to classify graphs constructed from clusters in the localization data. The clusters in each graph are each represented by discriminative features extracted from their constituent localizations, alongside the cluster coordinates, thereby retaining the original precision of the localizations.

We demonstrate this new pipeline on a model, open‐source SMLM dataset of DNA‐PAINT localizations from DNA origami nanostructures designed to resemble a selection of Digits and Letters.^[^ ^19^ ^]^ We present implementations of ClusterNet using both handcrafted features of individual clusters and features learnt with a neural network, achieving balanced classification accuracy of 99% across the dataset, improving on previous workflows.^[^ ^4^, ^18^, ^19^ ^]^ Further, we include and demonstrate the use of DL explainability algorithms to interpret the output of ClusterNet, for a data‐driven exploration of the results.^[^ ^5^ ^]^ Finally, we apply ClusterNet to a challenging new SMLM dataset from colorectal cancer tissue and compare performance using different data preprocessing algorithms and parameters. Localizations in SMLM FOVs of any spatial extent and complexity may be clustered and used as input to ClusterNet, allowing sample‐level classification from the entire point cloud.

## 2 Results

### 2.1 Classification Pipeline and Performance

**Figure 1:**
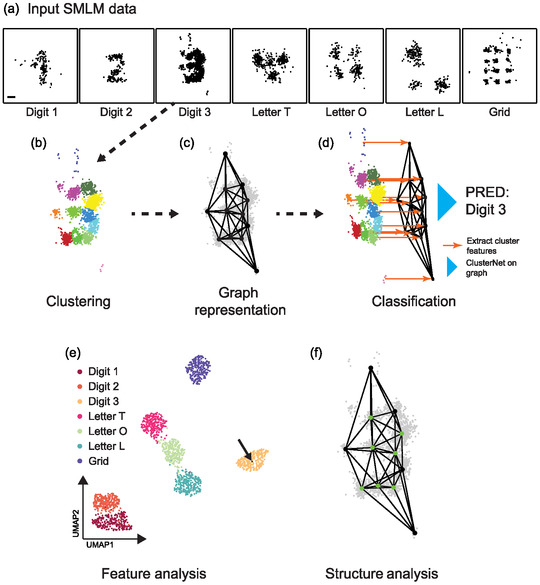
SMLM data classification pipeline. a) Example SMLM ROIs for each DNA origami structure, with GT labels below.^[^ ^19^ ^]^ Scale bar: 13 nm. b) Clusters of localizations from an ROI with GT Digit 3, colored by cluster identity. c) Graph representation of the ROI in (b) with localization nodes (gray dots), cluster nodes (black dots), and edges. d) Handcrafted features (in ClusterNet‐HCF) or automatically learnt features from DL (in ClusterNet‐LCF) are extracted for each cluster, and the graph composed from these clusters is passed to ClusterNet to give a final prediction (PRED). e) 2D representation of cluster or graph embeddings from UMAP, colored by GT (reserved test dataset, graph‐level features in this case, black arrow: ROI in (b–d). f) Important subgraph (green nodes) for classification, extracted using SubgraphX.

To classify 2D SMLM datasets using only the *xy* localization coordinates, we developed a pipeline that includes the supracluster structure using graph‐based DL models, and explores the important features and substructure used in classification via Uniform Manifold Approximation and Projection (UMAP) and SubgraphX.^[^ ^20^, ^21^ ^]^ We tested two novel classification models, ClusterNet‐handcrafted cluster features (HCF) and ClusterNet‐learned cluster features (LCF), which combine per‐cluster features of clustered localizations with the spatial arrangement of those clusters in a graph neural network (**Figure** **1**). ClusterNet‐HCF passes handcrafted per‐cluster features, for example, area and perimeter, through a graph neural network, ClusterNet. ClusterNet‐LCF uses an additional point‐based DL module, LocNet, to embed per‐cluster features for subsequent classification with ClusterNet, in a single trainable network.

We tested our pipeline on regions of interest (ROIs) from an existing DNA‐PAINT dataset acquired from DNA origami Digits and Letters (Figure 1a).^[^ ^19^ ^]^ We chose this dataset for the following reasons. It was large and contains point‐cloud data with the spatial coordinates and an estimate of their precision for each single‐molecule localization. It also had readily available ground truth (GT) labels making it well‐suited to training and validating a DL model. Finally, our pipeline could be compared against previous methods used to classify this dataset. The dataset additionally has features characteristic of most SMLM datasets, such as clustered localizations arranged in a nonrandom pattern and a wide range in the number of localizations and the localization precision between ROIs and classes (Table S1, Figure S1, Supporting Information). As found for more complex biological datasets, not all the localizations in each ROI contribute to the structure being analyzed or were as relevant to the GT label (e.g., background localizations or localizations far from any binding site).

Both ClusterNet‐HCF and ClusterNet‐LCF classified the data from the seven DNA origami structures in the dataset successfully. *k*‐means clustering was used to preprocess the localization data from the Digits and Letters (Methods), although any clustering algorithm could be applied. Both models achieved the maximum value of 1.00 for the area under the receiver operator curve (AUROC) for every class in the reserved test set. ClusterNet‐HCF outperformed ClusterNet‐LCF on the reserved test set (**Table** **1**) and on the training, validation and test folds (Table S2–4, Supporting Information).

**Table 1:** Classification performance on the DNA origami dataset. Recall values for ClusterNet‐HCF and ClusterNet‐LCF on the reserved test set.

	Digit 1	Digit 2	Digit 3	Letter T	Letter O	Letter L	Grid	Mean ± S.D.
ClusterNet‐HCF	0.99	0.98	1.00	0.96	0.99	0.99	1.00	**0.99 ± 0.01**
ClusterNet‐LCF	0.97	0.94	1.00	0.93	0.95	0.91	0.99	0.96 ± 0.03

ClusterNet‐HCF and ClusterNet‐LCF both outperformed previous results on the same dataset (**Table** **2**). In addition, the accuracy for previous methods (nanoTRON and point cloud registration) was calculated for classification within subsets (Digits/Grid or Letters) of the seven classes of DNA origami structure, whereas the accuracy of ClusterNet was from classification over all seven classes, which is a harder task.^[^ ^4^, ^18^ ^]^ The previous methods would be expected to give lower accuracies than reported in Table 2, if tested on the same task as ClusterNet.

**Table 2:** Classification performance compared with previous methods on the DNA origami dataset. Accuracy values for ClusterNet‐HCF and ClusterNet‐LCF on the reserved test set and closest comparisons with previous methods, nanoTRON and point cloud registration.^[^ ^4^, ^18^ ^]^

Model	Digits and Grid	Letters
nanoTRON	≈98% (n = 74 k)^a^^,^ b)	n/ac)
Point cloud registration	96.4% (n = 5000)^b^^,^ d)	89.0% (n = 600)^b^^,^ e)
ClusterNet‐HCF	**99.1%** (n = 960)^b^^,^ f)	**98.2%** (n = 720)^b^^,^ f)
ClusterNet‐LCF	97.4% (n = 960)b,, f)	93.1% (n = 720)^b^^,^ f)

a) The test dataset was reserved from a larger dataset, formed from 11‐fold expansion (via data augmentation) of 21 k unique ROIs.^[^ ^4^ ^]^

b) Accuracy for ClusterNet‐HCF and ClusterNet‐LCF included misclassifications between all Digits/Grid and Letters structures. Accuracy for point cloud registration included misclassifications only within either Digits/Grid or Letters subsets (not between them). nanoTRON was only tested on Digits/Grid structures.

c) The dataset included only Digits and Grid.

d) 5000 ROIs were randomly sampled from all ROIs over all Digits and Grid classes.

e) Accuracy when misfolded DNA origami Letter structures were classified as one of the three Letters (with no extra “misfolds” class), as in our method. 200 ROIs sampled per GT class.

f) Calculated from Table S5, S6, Supporting Information. 240 ROIs per GT class in reserved test dataset.

Classification performance (recall) was greater for the Digits and Grid than the Letters (Table 1). This was expected as there were misfolded DNA origami structures in the Letters dataset which did not resemble the intended Letter.^[^ ^18^ ^]^ This had less impact on the Digits and Grid, as the authors of the published dataset excluded some of the misfolded Digits and Grids in particle picking.^[^ ^18^ ^]^ There may be an additional contribution to this from the imbalance in the validation dataset used during training (fewer Letters than Digits and Grid), despite attempts to mitigate this with weighted random sampling of the training set based on the prevalence of each class.

### 2.2 Feature Analysis via UMAP

**Figure 2:**
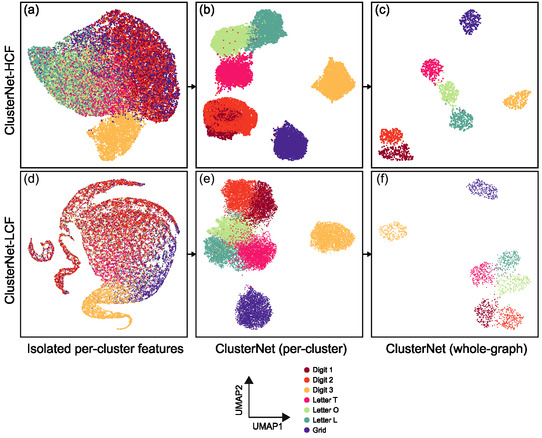
Feature analysis of SMLM ROI classification results. 2D feature representations (from UMAP) for the ROIs in the reserved test set, for a–c) ClusterNet‐HCF and d–f) ClusterNet‐LCF. The features incorporate larger structure from left to right: (a,d) isolated per‐cluster handcrafted or LocNet embedded features; (b,e) per‐cluster features after message passing but before global pooling in ClusterNet; and (c,f) whole‐graph features aggregated from ClusterNet.

The relative discriminative power of the handcrafted and deep features for the clusters was measured by comparing the separation of the clusters for each class in a 2D representation of the feature space generated using UMAP.^[^ ^20^ ^]^ Isolated per‐cluster features, with no incorporation of supracluster structure, were not able to separate the classes, except for the Digit 3 (**Figure** **2**a, d). However, the handcrafted features did slightly separate the Letters T, O, and L from Digits 1 and 2 and the Grid (Figure 2a). This reflected the differences in the DNA origami structures, where the Digits have continuous structures with no spaces between their binding sites, while the Letters have discrete structures, with groups of binding sites well‐separated from each other.^[^ ^18^ ^]^ The deep cluster features generated by LocNet did not separate the Digits and Letters but better separated the Grid, suggesting that they captured different aspects of the input data to the handcrafted features (Figure 2d). Digit 3 may have been separable at the per‐cluster level because it had a significantly different localization density from the other classes (Figure S1 and Table S1, Supporting Information). However, the remaining classes could not be separated based on differences in localization density alone, as they had a similar average number of localizations per ROI (Figure S1 and Table S1, Supporting information).

The cluster features generated by ClusterNet significantly improved separation into each class, showing the importance of considering the supracluster structure via information from neighboring clusters (Figure 2b,e). Similar to the handcrafted and LocNet cluster features, the Grid and Digit 3 classes were the most clearly separated for both models. The remaining Digits, 1 and 2, were separated from the Letters, although there was significant overlap within these two groups.

The whole‐graph features further improved the separation of the classes (Figure 2c,f). This showed the importance of moving from per‐cluster to whole‐graph features, highlighted by Digits 1 and 2 which changed from overlapping to well‐separated for ClusterNet‐HCF (Figure 2b,c). ClusterNet‐HCF had the most compact and well‐separated representations, reflecting its classification performance (Figure 2c). The Letters and Digits 1 and 2 were still assigned into two distinct groups.

### 2.3 Structure Analysis via SubgraphX

Different classes may be distinguished by the arrangement of some or all of their clusters (supracluster structure). We tested a method for identifying these structures by finding the important parts of a cluster graph for the classification of an SMLM ROI. SubgraphX searches for the most important subgraph for the graph classification,^[^ ^21^ ^]^ in this case a subset of clusters and their supracluster structure. We analyzed the classification results from ClusterNet‐HCF (the best performing model).

**Figure 3:**
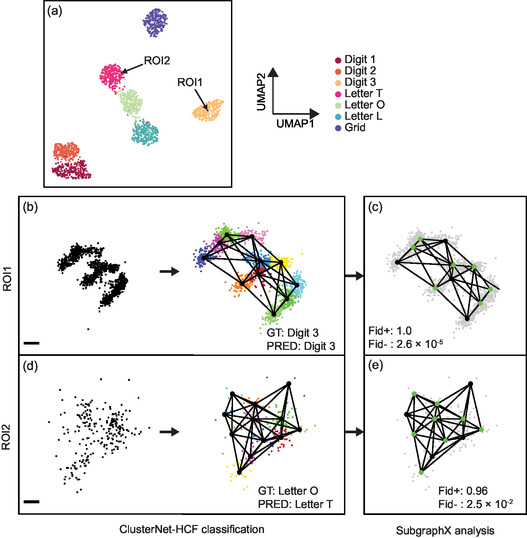
Structure analysis of SMLM ROI classification results. a) 2D whole‐graph feature representation (from UMAP) for ClusterNet‐HCF for the reserved test dataset ROIs, highlighting ROIs with whole‐graph features (from their classified cluster graph) closest to (ROI1) or furthest from (ROI2) their fellow class members. Classification and structure analysis of b,c) ROI1 and d, e) ROI2. (b, d): DNA‐PAINT localizations (scale: 13 nm) represented as a graph and classified by ClusterNet‐HCF (GT: ground truth, PRED: prediction). Localizations in graph representation colored by cluster identity. (c, e): SubgraphX results, showing important subgraph (supracluster structure) for class prediction (green nodes). Positive fidelity (Fid+) and negative fidelity (Fid−) measure the necessity and sufficiency respectively of the important subgraph (best performance: Fid+ = 1, Fid− = 0; worst performance: Fid+ = 0 and Fid− = 1).

The 2D representation of the whole‐graph feature space (Figure 2c) allowed us to choose ROIs closest to and furthest from the rest of their class members, measured by the distance to the centroid of the class features (e.g., **Figure** **3**a: ROI1, ROI2). After graph representation and classification (Figure 3b,d), SubgraphX returned the most important subgraph for the classification that it found (Figure 3c,e; for parameters see Experimental Section in Supporting Information). Positive and negative fidelity metrics (Fid+, Fid‐) measured the necessity and sufficiency, respectively, of the subgraph for the classification (Methods; best performance: Fid+ = 1, Fid− = 0; worst performance: Fid+ = 0 and Fid− = 1).^[^ ^22^ ^]^

An exemplary graph closest to its class members (Figure 3a: ROI1) was from a clear DNA‐PAINT example of a Digit 3, correctly predicted (Figure 3b). The subgraph identified by SubgraphX was both necessary and sufficient for the classification (Fid+: 1.0, Fid−: 2.6 × 10^−5^) and reflected the Digit 3 shape that would be expected (Figure 3c). An example furthest from its class members (Figure 3a: ROI2) may have been a misfolded item, as there only appear to be three well‐separated groups of localizations whereas the Letter O (the GT label) should have four (Figure 3d and Figure 1a).^[^ ^18^ ^]^ It was incorrectly predicted as the Letter T, which was reflected in its location near the other Letter Ts in the UMAP representation (Figure 3a, d). The subgraph extracted by SubgraphX was important for its classification (Fid+: 0.96, Fid−: 2.5 × 10^−2^) and resembled the Letter T structure (Figure 3e and Figure 1a). In general, the important subgraph for incorrectly classified ROIs did not appear to reflect the structure expected of the correct class (Figure S2, S3, Supporting Information), and a closer resemblance to the incorrectly predicted class could sometimes be discerned (Figure S2h,l,m,n and Figure S3b, Supporting Information). This suggests that this approach could allow us to begin to identify specific patterns in the supracluster structure that lead to classification results in SMLM datasets, including misclassifications (further results in Figure S4, Table S7, and Table S8, Supporting Information).

We also note that some clusters appeared outside of the designed DNA origami patterns, arising from imperfections in the original sample and raw data acquisition, influencing classification with ClusterNet (Figure S2a,e,h and Figure S3c, Supporting Information). SubgraphX showed that these spurious clusters were sometimes considered important in classification (Figure S2a,h, Supporting Information), also explaining some incorrect results (Figure S2h, Supporting Information).

### 2.4 Classification of Direct Stochastic Optical Reconstruction Microscopy (dSTORM) Biological Dataset

**Figure 4:**
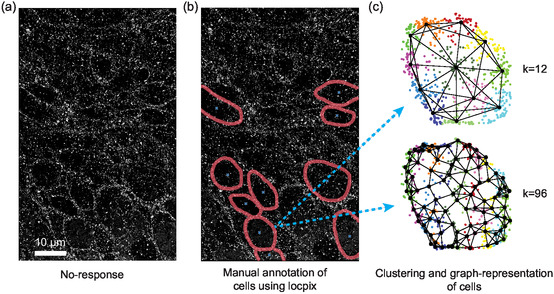
Classification of cells from advanced colorectal cancer patients by response to anti‐EGFR treatment. a) Example dSTORM image of EREG in a tissue section, from a patient that did not respond to treatment. b) EREG localizations for each cell, manually annotated using locpix (red lines: membrane, blue crosses: cell centers).^[^ ^40^ ^]^ c) Localizations for a cell from (b), clustered using *k*‐means for *k *= 12 or 96 clusters (color: cluster identity). Graph representations are overlaid (black dots: cluster nodes).

To determine if this pipeline can be used to classify more complex biological datasets and other SMLM techniques, we tested its ability to classify a dataset of dSTORM localizations for epiregulin (EREG) in tissue sections. The dataset was obtained by imaging cells (*n* = 163) stained for EREG in tissue microarrays (TMAs) from colorectal cancer patients (*n* = 23) (**Figure** **4**, Figure S5 and Table S9, Supporting Information).^[^ ^23^, ^24^ ^]^ The TMAs were obtained from patients classified by their response (no‐response vs. any‐response) to anti‐epidermal growth factor receptor (EGFR) treatment. This dataset is more challenging to classify, because the localizations have a lower signal‐to‐noise ratio than the Digits and Letters dataset, and the classes are not visually identifiable from reconstructed images of the cells.

The ClusterNet‐HCF pipeline successfully classified the cells. After changing the number of clusters identified in preprocessing from *k* = 12 to *k* = 96, the AUROC reached 0.70 ± 0.15 and balanced accuracy was 0.64 ± 0.14. This suggests that the spatial organization of the ligand EREG may help predict response to anti‐EGFR treatment.

We also investigated the effect of the clustering algorithm and parameters in preprocessing on classification results for this dataset. *k*‐means and density‐based spatial clustering of applications with noise (DBSCAN) were compared over a wide range of parameters (Table S10, Supporting Information). For *k*‐means, both AUROC and balanced accuracy improved when *k* = 12 was increased to *k* = 96, showing the importance of fine‐tuning the graph representation for each task. DBSCAN, with ε = 50 nm and minPts = 3, achieved the same AUROC as *k*‐means with *k* = 96. However, the variance in AUROC was greater between the test folds (greater uncertainty in performance on unseen test data) and balanced accuracy was decreased. We also found that *k*‐means was more robust to changes in parameters than DBSCAN, which had large differences in results for small changes to ε and minPts. Of note, removing some of the preprocessing steps such as filtering localizations and cells made little difference to results (see Supporting Information).

## 3 Discussion

We have demonstrated a pipeline for classifying SMLM fields of view, combining per‐cluster features extracted from the localization pattern with supracluster structure extracted via graph‐based DL (ClusterNet, Figure 1). Per‐cluster features may either be handcrafted and fully interpretable (e.g., cluster area, perimeter) or also obtained from DL (LocNet). We have also incorporated analysis of the features and structures learnt during classification. UMAP allowed investigation of cluster and supracluster structure feature embedding.^[^ ^20^ ^]^ It demonstrated that supracluster structure was required to separate the classes in the Digits and Letters dataset and revealed subpopulations such as Digit vs Letter structures without user specification (Figure 2). SubgraphX identified the important subgraphs (subsets of clusters in their supracluster arrangements) for classification (Figure 3).^[^ ^21^ ^]^

Combining handcrafted features (HCF) with ClusterNet (ClusterNet‐HCF) outperformed the model that extracted deep features at both the per‐cluster and whole‐graph scale (ClusterNet‐LCF) (Table 1, Figure 2c, f). This shows that incorporating known handcrafted features can boost classification performance, besides being more interpretable. In other SMLM datasets, deriving discriminative handcrafted features may be more difficult. We anticipate that generating cluster features via DL, as with LocNet, will be more useful in these cases. Further, extending ClusterNet‐LCF to include photophysical parameters as features of the localizations, or weighting localizations by these parameters in the calculation of handcrafted features, could allow the model to account for localization uncertainty.

Our classification pipeline outperformed previous methods applied to the Digits and Letters dataset (Table 2). One was designed for particle fusion and requires no training and minimal supervision,^[^ ^18^ ^]^ and the other was an image‐based model.^[^ ^4^ ^]^ It is likely that an alternative image‐based method could match our classification performance, but it would require large images (e.g. 1 pixel per nm) to incorporate the high precision of the SMLM data as the localizations are binned into pixels.^[^ ^14^, ^25^ ^]^ Such rendering does not scale well for larger ROIs or when moving from 2D to 3D SMLM data, as the size of the image increases exponentially.^[^ ^14^ ^]^ Instead, point‐based methods such as ours can be simply extended without a large increase in the size of the data representation. For ClusterNet, this would include changing the handcrafted features to characterize 3D rather than 2D shapes (e.g., area to volume), changing the dimension of the position vector for each localization and node from 2D to 3D and including 3D rotations during training to learn invariance to rotations not in the optical plane.

Analyzing high dimensional features of SMLM data can help to identify and investigate the underlying substructures that distinguish different SMLM data structures. This has mainly been restricted to visualization of the features in a lower‐dimensional space, via dimension reduction techniques such as UMAP.^[^ ^9^, ^10^, ^14^, ^26^ ^]^ For the Digits and Letters dataset, this revealed intraclass variability and identified misfolded structures by visualizing graphs closest to and furthest from the center of the class (Figure 3 and Figure S2, Supporting Information), without requiring an additional class to capture the misfolds.^[^ ^18^ ^]^

DL explainability algorithms can more directly identify the underlying substructures, by measuring their impact on the model's prediction. So far this has been used on SMLM data represented as images, but not on graph representations.^[^ ^5^ ^]^ For the Digits and Letters dataset, SubgraphX was used to identify the discriminative substructure in each FOV represented as a graph. In some cases, this was able to identify substructure that aligned with our knowledge of the GT (Figure 3). However, future work is required to make it more reliable, if this is to be applied to other SMLM data.

In this study, the pipeline was demonstrated on DNA‐PAINT and dSTORM data, but the pipeline could be applied to a broad range of SMLM data from other techniques that generate point‐cloud data (e.g., PALM) photoactivated localization microscopy. Our datasets had characteristics common to most SMLM data (e.g., being clustered or containing localizations that are less relevant to the classification task), making them a useful proof of concept. Moving between these different techniques or experimental conditions should not significantly affect handcrafted features that capture the overall shape and size of clusters. Learnt cluster features can adapt to any differences, as seen by the range of data types (e.g. 3D shapes, indoor and outdoor scenes etc.) that point‐based DL networks have been applied to.^[^ ^27^ ^]^ The model can then be retrained or adjusted to adapt to any remaining differences, for example, increasing the number of clusters improved the accuracy of ClusterNet for the dSTORM data from cells. To further improve the classification performance on the cells, other modifications to the pipeline could be tested, such as: including more handcrafted features, increasing the number of layers in the network, changing how the localizations and cells are filtered during preprocessing, or including another level of clusters (localizations, clusters, super‐clusters) to better capture larger structures in the data.

ClusterNet can be applied to complex biological samples as we show here. ClusterNet was able to classify tumor samples into response and no response to treatment. The drop in classification performance compared to the Digits and Letters reflects the challenge of classifying complex biological samples, with lower signal‐to‐noise ratio and with fewer ROIs available for model testing. Results on the tumor samples also highlighted factors that may impact classification performance, such as how the data is preprocessed or the choice of graph representation and clustering technique. Furthermore, the TMAs contain multiple cell types, some of which may not change their expression or organization of EREG in cancer (e.g., lymphocyte, enterocyte, etc.), and which could not be identified from the dSTORM data. These cells could therefore not be excluded from the annotation stage, which is likely to have affected the overall classification performance metrics. With further development, this pipeline could be used to differentiate between disease conditions or biological states from SMLM data on cells and tissues, refining current techniques in pathology that use ligand expression.^[^ ^28^, ^29^, ^30^ ^]^ ClusterNet can therefore contribute to realizing the use of SMLM data as a new modality in characterization of phenotype, classification of disease progression and prediction of response to treatment.^[^ ^31^, ^32^, ^33^, ^34^ ^]^ We also provide a new route to increased understanding of disease and other biological processes through downstream feature and structure analysis.

## 4 Experimental Section

4.1

4.1.1

##### Digits and Letters Dataset

To develop and test our pipeline we used the open‐source Digits and Letters dataset from.^[^ ^19^ ^]^ Further details on how these datasets were acquired can be found at^[^ ^4^ ^]^ for the Digits and Grid and at^[^ ^18^ ^]^ for the Letters. The dataset comprises 22 047 SMLM ROIs from DNA‐PAINT data acquired from DNA origami structures, with one structure per ROI. This included Digits (4155 × Digit 1, 4943 × Digit 2, 2541 × Digit 3), Letters (1161 × Letter L, 991 × Letter T, 560 × Letter O), and a 3 × 4 grid (7696 × Grid), imaged separately per class.^[^ ^4^, ^18^ ^]^ The number of localizations per ROI and localization uncertainty for each class are visualized and characterized in Figure S1 and Table S1, Supporting Information.

##### Tumor Dataset from the PICCOLO Trial

PICCOLO was a randomized phase III trial of second‐line irinotecan with or without panitumumab (anti‐EGFR therapy) in advanced colorectal cancer.^[^ ^23^, ^24^ ^]^ As part of the trial, pretreatment tumor samples were collected from each patient (mainly from the primary tumors but with ≈5% from metastases). Best response by Response Evaluation Criteria In Solid Tumors (RECIST) criteria within 1‐year follow‐up from randomization was recorded.^[^ ^24^, ^35^ ^]^

In our study, we imaged cells in tumor cores from the preconstructed formalin‐fixed paraffin‐embedded (FFPE) TMA blocks from PICCOLO, preferred over whole slides to minimize time identifying tumor regions and increase throughput. Only patients who received anti‐EGFR therapy (panitumumab arm) and tested wild‐type (WT) for confounding genetic mutations were included (mutations in *KRAS* codons 12, 13, 61, and 146; *NRAS* codons 12, 13, and 61; and *BRAF* codon 1799 T > A). Patients with *BRAF*‐mutant tumors were excluded because anti‐EGFR therapy is less effective for this subgroup.^[^ ^36^ ^]^ The best response variables (from RECIST) were grouped into any‐response (complete response, partial response) and no‐response (radiological progression, clinical progression, death), with patients that only achieved stable disease excluded to simplify the problem.

##### Sample Preparation and SMLM Imaging for the Tumor Dataset

3 μm sections were cut from each preconstructed FFPE TMA block and placed on APES‐coated 1.5 H coverslips. Coverslips were placed on a hotplate at 70 °C for 30 min and then inside a pressure cooker with Reveal Decloaker (pH 6.0) for antigen retrieval. At room temperature, samples were then quenched with ammonium chloride for 15 min, permeabilized with 0.5% Triton‐X in (PBS) phosphate buffered saline for 1 h and blocked with 3% bovine serum albumin (BSA) plus 20% donkey serum for 1 h. Samples were then incubated with Roche RTU anti‐EREG antibody (SP326: rabbit monoclonal, Roche) overnight at 4 °C and then with donkey anti‐rabbit Alexa 647 for 1 h at room temperature.

Each section contained samples from up to ≈30 patients, with three tumor cores (preselected from the region of highest tumor) per patient. For each patient included in this study, the core with the highest tumor content (greatest area of anti‐EREG staining in widefield scans) was selected for imaging. One FOV was imaged at the region of greatest staining within the core.

To image the samples, we performed total internal reflection fluorescence microscopy dSTORM imaging using a commercial system (Nanoimager, ONI, UK) and a 100 × 1.4 NA oil‐immersion objective lens with a 50 × 80 μm FOV. Samples were bathed in STORM buffer (B‐cubed buffer, ONI, BCA0017). Using an exposure time of 30 ms, 10 000 frames were acquired using 640 nm laser excitation at 80% power of the maximum excitation output of the Nanoimager. 2D localization of fluorescence emission events was performed while imaging using NimOS (ONI).

Drift correction, filtering, and temporal grouping for each FOV were performed using CODI (COllaborative DIscovery platform from ONI; https://oni.bio/nanoimager/software/codi‐software/). Localizations with >30 000 photons, with a standard deviation of the fitted point spread function (PSF) <75 nm or >200 nm, with a *p*‐value for the fitted PSF above 0.01 or with a localization precision >25 nm were removed. Localizations within 60 nm and no more than two frames apart were grouped, removing those that existed for longer than five frames. The data in its proprietary format was then converted into an Apache Parquet file, a column‐oriented data format which can be more efficient for querying and storing than.csv files (https://parquet.apache.org/). For each localization, the channel, frame number, and *xy* coordinates were stored.^[^ ^37^ ^]^

##### Preprocessing and Clustering the Digits and Letters Dataset

Each ROI was preprocessed and clustered in preparation for feature extraction and graph representation as follows. The point cloud was initially partitioned into clusters following a similar approach to PointTransformer V2.^[^ ^38^ ^]^ First, the *xy* localization coordinates for each ROI were converted from a MATLAB to an Apache Parquet file and labeled in the metadata according to the character they represent. This gave seven classes (Digit 1, Digit 2, Digit 3, Letter T, Letter O, Letter L, or Grid). Next, *k*‐means clustering was applied to each ROI with *k* set to 12 to ensure every well‐separated group of DNA‐PAINT binding sites was recovered (Grid had 12 well‐separated binding sites; Digits had more binding sites, but not well‐separated). Clusters with two or fewer localizations were discarded to allow future calculation of the convex hull and principal components. We chose *k*‐means clustering over DBSCAN, as DBSCAN requires careful tuning of two hyperparameters rather than one and risks dropping many important localizations considered as noise.^[^ ^39^ ^]^

##### Cell Annotation, Preprocessing, and Clustering

Cells within FOVs were annotated and their localizations extracted using locpix.^[^ ^40^ ^]^ First, the unfiltered localizations were rendered into a histogram to facilitate annotation. The membranes of cells with low intensity at their center and high intensity at their membrane were annotated, generating a membrane mask. Annotations were either closed with themselves or the edge of the FOV. Cells were ≈5–15 μm in diameter and could be at the edge of the FOV (only partially visible). The cell‐containing region was generated by flood‐filling the membrane mask at seed locations manually placed at the cell centers. The watershed algorithm was then applied to the membrane mask, over the cell‐containing region, to generate the individual cell masks. Having first checked these annotations still correctly overlaid the higher quality localizations (with drift correction, filtering, and temporal grouping), the cell masks and membrane annotations were used to extract the higher quality localizations for each cell and label each localization according to its location (membrane or interior). All localizations not part of a cell were removed. Cells with fewer than 500 localizations, or with fewer than five interior or membrane localizations, were removed. The *xy* localization coordinates for each cell were saved as an Apache Parquet file with a label in the metadata for the treatment response (any‐response or no‐response). This gave 163 cells (no‐response: 117, any‐response: 46) from 23 patients (no‐response: 18, any‐response: 5) in which the numbers of cells per patient were unequal, ranging from 1–31 cells per patient (Figure S5, Supporting Information).

Each cell was clustered in preparation for feature extraction and graph representation. We trialed *k*‐means clustering, with *k* = 12, 24, 48, 72, 96, 120, or 144, and DBSCAN clustering, with epsilon, *ε* = 50 nm, 75 nm, or 100 nm and minimum samples, minPts = 3, 5, or 7. The DBSCAN parameters were set to similar values as used in previous studies to identify clusters of EGFR in SMLM data.^[^ ^40^, ^41^ ^]^ Clusters with two or fewer localizations were discarded to allow calculation of the convex hull and principal components.

##### Handcrafted Feature Extraction

Eight handcrafted features were calculated for each cluster. First, the number of localizations per‐cluster (count), the mean squared distances of localizations within the cluster from the cluster centroid (radius of gyration squared) and the perimeter from its convex hull were calculated. Next, principal component analysis was used to calculate the variance of the clusters along the two principal components, λ_0_ and λ_1,_ where $\lambda_{0}>\lambda_{1}$. These were used to calculate linearity ($\frac{\sqrt{\lambda_{0}}\;-\;\sqrt{\lambda_{1}}\;}{\sqrt{\lambda_{0}}\;}$), planarity $(\sqrt{\frac{\lambda_{1}}{\lambda_{0}})}$, length $\left(2.35\times \lambda_{0}\right),$ and area $\left(2.35^{2}\times \lambda_{0}\lambda_{1}\right)$of each cluster (the full width at half maximum of a Gaussian is given by 2.35 times the standard deviation).^[^ ^42^, ^43^ ^]^ In 3D, planarity is given by $\frac{\sqrt{\lambda_{1}}-\sqrt{\lambda_{2}}}{\sqrt{\lambda_{0}}}$, where λ_2_ is the third principal component.^[^ ^42^ ^]^ The data were 2D in this study, so λ_2_ = 0 and linearity = 1−planarity, which made one of linearity or planarity redundant. However, nothing is lost by including the redundant features in 2D, except a small increase in memory usage and training time. Finally, density for each cluster was calculated by dividing count by area.

##### Graph Construction

Each SMLM ROI (Digits and Letters) or cell (tumor dataset) was represented as a graph using PyTorch Geometric.^[^ ^44^ ^]^ Each graph contained localization and cluster nodes (from clustering as described), where each localization node belonged to its cluster node and undirected edges connected each cluster node to itself and its nearest five neighbors. The position of each localization node was given by its coordinates and the position of each cluster node was given by the center of mass of its constituent localizations. *xy* node positions were normalized to between −1 and 1, *x* $\to \frac{\text{2( x}-\text{min}\left(x\right))}{\max\left(x_{range},y_{range}\right)}-1$ and *y* $\to \frac{\text{2( y}-\text{min}\left(y\right))}{\max\left(x_{range},y_{range}\right)}-1$, where the minimum and range were measured over the parent graph. Cluster nodes initially had either no features or the eight handcrafted features depending on the downstream model (learnt or handcrafted features). When present, these features, *h*, were normalized to between zero and one, $h\to \frac{h-\text{min}(h)}{\text{max}(h)-\text{ min}(h)}$, where the minimum and maximum values were measured over the whole training set for the relevant dataset. The localization nodes had no input features.

##### Data Partitions

For the Digits and Letters dataset, 20 367 graphs (Digit 1: 3915, Digit 2: 4703, Digit 3: 2301, Letter L: 921, Letter T: 751, Letter O: 320, Grid: 7456) were used for five‐fold cross‐validation. A further 240 graphs from each class formed a reserved test set. The five different splits in cross‐validation each contained a training (64%), validation (16%), and test set (20%, nonoverlapping between splits). For each split, the training, validation, and test set each had the same proportion of classes as the overall cross‐validation dataset.

For the cell dataset, the cells were partitioned for five‐fold cross‐validation of model performance. The cell dataset was first divided into five folds (groups of cells). Each fold was used for testing, with the remaining four folds used for training. During training, 20% of this training set formed a validation set, where the ratio of any‐response to no‐response cells in the validation set was the same as in the training set, or as close as possible. This was used to monitor performance on data not used to train the model. The cells from a single patient could only be in one of the folds, to ensure that none of the models were trained and tested on cells from the same patient. Further, as best as possible, the folds had the same ratio of any‐response to no‐response cells as in the overall dataset.

##### Model Architecture

Two different neural network models were developed to classify each graph, ClusterNet‐HCF and ClusterNet‐LCF. ClusterNet‐HCF passed HCF, as described, and the positions of the cluster nodes, through a novel graph neural network, ClusterNet. ClusterNet‐LCF instead generated cluster features via DL using an additional point‐based module, LocNet, and passed them, with cluster position, through ClusterNet in a single network. Brief descriptions of the models are given below, with further details in Methods and Figure S6 in Supporting Information.

LocNet acted on each cluster independently, taking the constituent localization node positions, $p\in {\mathbb{R}}^{2}$, as input and embedding the localizations into a feature vector (length eight) for each cluster using PointTransformer v1.^[^ ^45^, ^46^ ^]^ The feature vector was chosen to be length eight to allow a fair comparison between ClusterNet‐HCF, which used an eight‐dimensional input feature, and ClusterNet‐LCF. Increasing the dimension of this feature vector may allow ClusterNet‐LCF to represent and classify more complex structures. Output cluster node features from LocNet were then scaled to between 0 and 1 using the sigmoid function.

ClusterNet acted on the graph of cluster nodes with their feature vectors and their *xy* position. It was composed of four message passing layers with PointTransformer convolutions, a global maximum pooling layer over the cluster features, resulting in a feature vector for the graph, and a final linear layer, generating a class prediction. When classifying the cells, the number of output channels for the ClusterNet was changed from 7 to 2 (Figure S6, Supporting information).

##### Training Procedure

For each split in five‐fold cross‐validation, the model was trained on the training dataset for 100 epochs using the ADAM optimizer with a learning rate of 0.001, a weight decay of 0.0001, and a batch size of 128 for the Digits and Letters or eight for the cells.^[^ ^47^ ^]^ During training, a weighted random sampler that oversampled from the minority class and undersampled from the majority class was used to encourage equal performance across the classes. Further, random rotations in the *xy* plane were applied to the graphs for data augmentation. For each graph, the model outputted the log probability for each class and the negative log‐likelihood loss was calculated. After each epoch, the model was evaluated on the validation set. The model that gave the lowest loss on the validation set over all the epochs was chosen as the best model. Training for 100 epochs on a NVIDIA GeForce RTX 2060 with 6 GB RAM took ≈1.5 h for the Digits and Letters dataset and ≈5 min for the cell dataset. ClusterNet‐LCF was trained in an end‐to‐end manner, meaning that LocNet and ClusterNet were trained together as a single network.

##### Evaluation Procedure

The best model for each split was evaluated on the test set for each split. For evaluation, each graph (without random rotation) was classified according to the highest probability class from the model. For the Digits and Letters the predictions for each class were evaluated using recall and combined into a single metric for all the classes using the arithmetic mean (balanced accuracy).^[^ ^48^ ^]^ For the cell dataset, the predictions for both classes were evaluated using balanced accuracy for binary classification.^[^ ^48^ ^]^ The probabilities for each class were evaluated using the AUROC.

##### Evaluation on the Reserved Test Set for the Digits and Letters Dataset

The performance of ClusterNet‐HCF and ClusterNet‐LCF were ultimately compared on the reserved test set. First, the entire dataset (excluding the reserved test set) was split into a training (80%) and validation (20%) set, by randomly taking ≈20% of each class into the validation set. Next, each model was trained on the training set and saved when the loss was lowest on the validation set. These models were then evaluated on the reserved test set following the evaluation procedure above.

##### Feature Analysis via UMAP

We used UMAP to visualize the per‐cluster and whole‐graph features and to explore if they separated the classes. Four sets of features were visualized: HCF, cluster features embedded by LocNet, cluster features after the fourth message passing layer of ClusterNet, and whole‐graph features after the final maximum pooling layer of ClusterNet. The handcrafted and LocNet features only consider the structure at cluster‐level and smaller and are later referred to as isolated per‐cluster features. The cluster features from ClusterNet have a larger receptive field incorporating information from neighboring clusters. Finally, the whole‐graph features from ClusterNet pool information over all constituent clusters. In all cases, features were normalized by subtracting the mean and dividing by the variance of each feature independently, measured over the entire dataset excluding the reserved test set. UMAP generated a lower‐dimensional (2D) representation of the features, with 20 neighbors for each feature vector and 0.5 minimum distance in the lower‐dimensional space. UMAP plots can be visualized interactively, displaying the parent ROI, GT, and prediction.

##### Structure Analysis via SubgraphX

SubgraphX was used to identify structures in the graphs constructed from the cluster nodes (cluster graphs) and their features (handcrafted or embedded by LocNet) that were important for the classification.^[^ ^21^ ^]^ SubgraphX was preferred over similar methods due to its high performance in non‐SMLM graph data benchmarks.^[^ ^49^ ^]^ SubgraphX searches for the most important connected subgraph in the cluster graph by feeding subgraphs induced by different sets of nodes into ClusterNet and measuring their relative contribution to the model's prediction (see methods section in Supporting Information). The optimal subgraph was required to have no more than eight nodes, and the number of rollouts was increased from 20 (default value) to 100, to minimize instability of the prediction (further details and parameter values in methods section in Supporting Information).

Positive and negative fidelity scores measured the necessity and sufficiency, respectively, of the optimal subgraph (subset of cluster nodes) for the prediction.^[^ ^22^ ^]^ They calculated the difference between the probability of the predicted class when the whole graph was fed into the model, and when the graph minus the subgraph (positive fidelity) or only the subgraph (negative fidelity) was fed into the model.^[^ ^50^, ^51^, ^52^, ^53^ ^]^ Best performance was given by a positive fidelity of one and negative fidelity of zero, and worst performance by positive fidelity of zero and negative fidelity of one.

## Code Availability

All code used for analysis was written in Python and is available at https://github.com/oubino/locpix_points/tree/v0.0.1, with latest developments available at https://github.com/oubino/locpix_points. We highlight particular dependencies in Methods, Supporting Information.

## Ethics Statement

Ethical approval for the work involving human tissue was granted by the North East York Research Ethics Committee (08/H0903/62). The study includes patients who, at the point of consent for the PICCOLO trial, had agreed to the use of their archival tissue in future research and for whom adequate stored formalin‐fixed, paraffin‐embedded (FFPE) tumor tissue remained. The ethics for PICCOLO trial consent and use in research is COREC 06/Q0906/38.

## Supporting Information

Supporting Information is available from the Wiley Online Library or from the author.

## Conflict of Interest

The authors declare no conflict of interest.

## Supporting information

Supplementary Material

## Data Availability

The original Digits and Letters dataset is available at https://doi.org/10.4121/14074091 and the files for reproducing the results on this dataset including the processed data and models can be found at https://doi.org/10.5281/zenodo.14246303. The cell dataset can be found at https://doi.org/10.5281/zenodo.17019520.

## References

[1] I. M. Khater , I. R. Nabi , G. Hamarneh , Patterns N Y 2020, 1, 100038. 33205106 10.1016/j.patter.2020.100038 PMC7660399

[2] S. Hugelier , P. L. Colosi , M. Lakadamyali , Annu. Rev. Biophys. 2023, 52, 139. 37159293 10.1146/annurev-biophys-111622-091212 PMC11268362

[3] I. R. Nabi , B. Cardoen , I. M. Khater , G. Gao , T. H. Wong , G. Hamarneh , J. Cell Biol. 2024, 223, 10.1083/jcb.202311073. PMC11169916 38865088

[4] A. Auer , M. T. Strauss , S. Strauss , R. Jungmann , Bioinformatics 2020, 36, 3620. 32145010 10.1093/bioinformatics/btaa154 PMC7267816

[5] D. Carnevali , L. Zhong , E. Gonzalez-Almela , C. Viana , M. Rotkevich , A. Wang , D. Franco-Barranco , A. Gonzalez-Marfil , M. V. Neguembor , A. Castells-Garcia , I. Arganda-Carreras , M. P. Cosma , Nat. Mach. Intell. 2024, 6, 1021. 39309215 10.1038/s42256-024-00883-x PMC11415298

[6] I. M. Khater , F. Meng , T. H. Wong , I. R. Nabi , G. Hamarneh , Sci. Rep. 2018, 8, 9009. 29899348 10.1038/s41598-018-27216-4 PMC5998020

[7] Y. L. Li , I. M. Khater , C. Hallgrimson , B. Cardoen , T. H. Wong , G. Hamarneh , I. R. Nabi , Adv. Intell. Syst. 2024, 7, 2400521. 40726957 10.1002/aisy.202400521 PMC12291151

[8] J. S. H. Danial , A. J. Garcia-Saez , Nat. Methods 2019, 16, 711. 31263253 10.1038/s41592-019-0472-1 PMC6675600

[9] S. W. B. Bender , M. W. Dreisler , M. Zhang , J. Kaestel-Hansen , N. S. Hatzakis , Nat. Commun. 2024, 15, 1763. 38409214 10.1038/s41467-024-46106-0 PMC10897458

[10] S. Hugelier , Q. Tang , H. H. Kim , M. T. Gyparaki , C. Bond , A. N. Santiago-Ruiz , S. Porta , M. Lakadamyali , Nat. Methods 2024, 21, 1909. 39256629 10.1038/s41592-024-02414-3 PMC11466814

[11] I. M. Khater , F. Meng , I. R. Nabi , G. Hamarneh , Bioinformatics 2019, 35, 3468. 30759191 10.1093/bioinformatics/btz113 PMC6748737

[12] T. H. Wong , I. M. Khater , B. Joshi , M. Shahsavari , G. Hamarneh , I. R. Nabi , Sci. Rep. 2021, 11, 7810. 33833286 10.1038/s41598-021-86770-6 PMC8032680

[13] T. H. Wong , I. M. Khater , C. Hallgrimson , Y. L. Li , G. Hamarneh , I. R. Nabi , J. Cell Sci. 2025, 138, JCS263570. 39865933 10.1242/jcs.263570

[14] I. M. Khater , S. T. Aroca-Ouellette , F. Meng , I. R. Nabi , G. Hamarneh , PLoS One 2019, 14, e0211659. 31449531 10.1371/journal.pone.0211659 PMC6709882

[15] Y. Fan , S. É.B. Faisan , F. Zwettler , M. Sauer , D. Fortun 2021 IEEE 18th Inter. Symp. on Biomedical Imaging (ISBI) 858-8622021, Nice, France 2021.

[16] L. A. Saavedra , A. Mosqueira , F. J. Barrantes , Nanoscale 2024, 16, 15308. 39082742 10.1039/d4nr01944j

[17] V. J. Sabinina , M. J. Hossain , J. K. Heriche , P. Hoess , B. Nijmeijer , S. Mosalaganti , M. Kueblbeck , A. Callegari , A. Szymborska , M. Beck , J. Ries , J. Ellenberg , Mol. Biol. Cell. 2021, 32, 1523. 34191541 10.1091/mbc.E20-11-0728 PMC8351745

[18] T. Huijben , H. Heydarian , A. Auer , F. Schueder , R. Jungmann , S. Stallinga , B. Rieger , Nat. Commun. 2021, 12, 3791. 34145284 10.1038/s41467-021-24106-8 PMC8213809

[19] T. A. P. M. Huijben , H. Heydarian , B. Rieger , S. Stallinga , R. Jungmann , F. Schueder , A. Auer 4TU.ResearchData 2021, 10.4121/14074091.v1. PMC8213809 34145284

[20] L. McInnes , J. Healy J. Melville UMAP: Uniform Manifold Approximation And Projection For Dimension Reduction. arXiv:1802.03426 2018. 10.48550/arXiv.1802.03426

[21] H. Yuan , H. Yu , J. Wang , K. Li S. Ji On Explainability Of Graph Neural Networks Via Subgraph Explorations. arXiv:2102.05152 (2021). 10.48550/arXiv.2102.05152

[22] K. Amara , Z. Ying , Z. Zhang , Z. Han , Y. Zhao , Y. Shan , U. Brandes , S. Schemm , C. Zhang GraphFramEx: Towards Systematic Evaluation of Explainability Methods for Graph Neural Networks. arXiv:2206.09677, 10.48550/arXiv.2206.09677.

[23] G. Middleton , S. Brown , C. Lowe , T. Maughan , S. Gwyther , A. Oliver , S. Richman , D. Blake , V. Napp , H. Marshall , J. Wadsley , N. Maisey , I. Chau , M. Hill , S. Gollins , S. Myint , S. Slater , J. Wagstaff , J. Bridgewater , M. Seymour , Eur. J. Cancer 2013, 49, 3507. 23953030 10.1016/j.ejca.2013.06.017

[24] M. T. Seymour , S. R. Brown , G. Middleton , T. Maughan , S. Richman , S. Gwyther , C. Lowe , J. F. Seligmann , J. Wadsley , N. Maisey , I. Chau , M. Hill , L. Dawson , S. Falk , A. O’Callaghan , K. Benstead , P. Chambers , A. Oliver , H. Marshall , V. Napp , P. Quirke , Lancet Oncol. 2013, 14, 749. 23725851 10.1016/S1470-2045(13)70163-3 PMC3699713

[25] D. Baddeley , M. B. Cannell , C. Soeller , Microsc. Microanal. 2010, 16, 64. 20082730 10.1017/S143192760999122X

[26] H. Verdier , F. Laurent , A. Casse , C. L. Vestergaard , C. G. Specht , J. B. Masson , PLoS Comput. Biol. 2023, 19, e1010088. 36730436 10.1371/journal.pcbi.1010088 PMC9928078

[27] S. A. Bello , S. Yu , C. Wang , J. M. Adam , J. Li , Remote Sens. 2020, 12.

[28] J. F. Seligmann , F. Elliott , S. D. Richman , B. Jacobs , G. Hemmings , S. Brown , J. H. Barrett , S. Tejpar , P. Quirke , M. T. Seymour , JAMA Oncol. 2016, 2, 633. 26867820 10.1001/jamaoncol.2015.6065

[29] C. J. M. Williams , F. Elliott , N. Sapanara , F. Aghaei , L. Zhang , A. Muranyi , D. Yan , I. Bai , Z. Zhao , M. Shires , H. M. Wood , S. D. Richman , G. Hemmings , M. Hale , D. Bottomley , L. Galvin , C. Cartlidge , S. Dance , C. M. Bacon , L. Mansfield , K. A. Sudan , K. Lambert , I. Bibby , S. E. A. Montazeri , N. Kipling , K. Hughes , S. S. Cross , A. Dewdney , Clin. Cancer Res. 2023, 29, 4153. 37363997 10.1158/1078-0432.CCR-23-0859 PMC10570673

[30] C. J. M. Williams , J. F. Seligmann , F. Elliott , M. Shires , S. D. Richman , S. Brown , L. Zhang , S. Singh , J. Pugh , X. M. Xu , A. Muranyi , C. Guetter , A. Lorsakul , U. Kurkure , Z. Zhao , J. Martin , X. Wang , K. Nguyen , W. W. Liu , D. Yan , N. P. West , J. H. Barrett , M. Barnes , I. Bai , M. T. Seymour , P. Quirke , K. Shanmugam , Clin. Cancer Res. 2021, 27, 3422. 33888518 10.1158/1078-0432.CCR-21-0120

[31] A. L. Maddox , M. S. Brehove , K. R. Eliato , A. Saftics , E. Romano , M. F. Press , J. Mortimer , V. Jones , D. Schmolze , V. L. Seewaldt , T. Jovanovic-Talisman , Cancers Basel 2022, 14, 2795. 35681773 10.3390/cancers14112795 PMC9179327

[32] J. Xu , H. Ma , H. Ma , W. Jiang , C. A. Mela , M. Duan , S. Zhao , C. Gao , E. R. Hahm , S. M. Lardo , K. Troy , M. Sun , R. Pai , D. B. Stolz , L. Zhang , S. Singh , R. E. Brand , D. J. Hartman , J. Hu , S. J. Hainer , Y. Liu , Nat. Commun. 2020, 11, 1899. 32313005 10.1038/s41467-020-15718-7 PMC7171144

[33] J. Xu , X. Sun , K. Kim , R. M. Brand , D. Hartman , H. Ma , R. E. Brand , M. Bai , Y. Liu Sci. Adv. 8, eabm8293 2022. 35245126 10.1126/sciadv.abm8293 PMC8896800

[34] Y. Wang , J. Gao , X. Guo , T. Tong , X. Shi , L. Li , M. Qi , Y. Wang , M. Cai , J. Jiang , C. Xu , H. Ji , H. Wang , Cell Res. 2014, 24, 959. 25001389 10.1038/cr.2014.89 PMC4123299

[35] E. A. Eisenhauer , P. Therasse , J. Bogaerts , L. H. Schwartz , D. Sargent , R. Ford , J. Dancey , S. Arbuck , S. Gwyther , M. Mooney , L. Rubinstein , L. Shankar , L. Dodd , R. Kaplan , D. Lacombe , J. Verweij , Eur. J. Cancer 2009, 45, 228. 19097774 10.1016/j.ejca.2008.10.026

[36] S. Stintzing , K. Heinrich , D. Tougeron , D. P. Modest , I. Schwaner , J. Eucker , R. Pihusch , M. Stauch , F. Kaiser , C. Kahl , M. Karthaus , C. Muller , C. Burkart , A. Reinacher-Schick , S. Kasper-Virchow , L. Fischer von Weikersthal , B. Krammer-Steiner , G. W. Prager , J. Taieb , V. Heinemann , J. Clin. Oncol. 2023, 41, 4143. 37352476 10.1200/JCO.22.01420

[37] ONI . How To Guide: Filtering Tool, https://help.codi.bio/portal/en/kb/articles/how-to-guide-filtering-tool 2025.

[38] X. Wu , Y. Lao , L. Jiang , X. Liu H. Zhao Point Transformer V2: Grouped Vector Attention And Partition-Based Pooling. arXiv:2210.05666 2022. 10.48550/arXiv.2210.05666

[39] M. Ester , H.-P. Kriegel , J. Sander , X. Xu , Proc. of the Second Inter. Conf. on Knowledge Discovery and Data Mining 226–231, Portland, Oregon, AAAI Press 1996.

[40] O. Umney , J. Leng , G. Canettieri , N. A. R. Galdo , H. Slaney , P. Quirke , M. Peckham , A. Curd , J. Microsc 2024, 296, 214. 39092628 10.1111/jmi.13349

[41] K. Jahnke , N. Struve , D. Hofmann , M. J. Gote , M. Bach , M. Kriegs , M. Hausmann , Nanoscale 2024, 16, 15240. 39073345 10.1039/d4nr01570c

[42] J. Demantké , C. Mallet , N. David , B. Vallet , Int. Arch. Photogramm. Remote Sens. Spatial Inform. Sci. 2011, 3812, 97.

[43] D. Baddeley , I. Jayasinghe , L. Lam , S. Rossberger , M. B. Cannell , C. Soeller , Proc. Natl. Acad. Sci. USA 2009, 106, 22275. 20018773 10.1073/pnas.0908971106 PMC2799702

[44] M. Fey J. E. Lenssen Fast Graph Representation Learning With PyTorch Geometric. arXiv e-prints, arXiv:1903.02428 2019. 10.48550/arXiv.1903.02428.

[45] C. R. Qi , H. Su , K. Mo L. J. Guibas PointNet: Deep Learning On Point Sets For 3D Classification And Segmentation. arXiv:1612.00593 2016. 10.48550/arXiv.1612.00593.

[46] H. Zhao , L. Jiang , J. Jia , P. Torr , V. Koltun , 2021 IEEE/CVF Inter. Conf. on Computer Vision (ICCV) 16239-16248, Montreal, Canada 2021.

[47] D. P. Kingma , J. Ba , 3rd Inter. Conf. on Learning Representations (Eds: Yoshua Bengio , Yann LeCun , San Diego, CA, USA 2015.

[48] L. Maier-Hein , A. Reinke , P. Godau , M. D. Tizabi , F. Buettner , E. Christodoulou , B. Glocker , F. Isensee , J. Kleesiek , M. Kozubek , M. Reyes , M. A. Riegler , M. Wiesenfarth , A. E. Kavur , C. H. Sudre , M. Baumgartner , M. Eisenmann , D. Heckmann-Notzel , T. Radsch , L. Acion , M. Antonelli , T. Arbel , S. Bakas , A. Benis , M. B. Blaschko , M. J. Cardoso , V. Cheplygina , B. A. Cimini , G. S. Collins , K. Farahani , L. Ferrer , A. Galdran , B. van Ginneken , R. Haase , D. A. Hashimoto , M. M. Hoffman , M. Huisman , P. Jannin , C. E. Kahn , D. Kainmueller , B. Kainz , A. Karargyris , A. Karthikesalingam , F. Kofler , A. Kopp-Schneider , A. Kreshuk , T. Kurc , B. A. Landman , G. Litjens , A. Madani , K. Maier-Hein , A. L. Martel , P. Mattson , E. Meijering , B. Menze , K. G. M. Moons , H. Muller , B. Nichyporuk , F. Nickel , J. Petersen , N. Rajpoot , N. Rieke , J. Saez-Rodriguez , C. I. Sanchez , S. Shetty , M. van Smeden , R. M. Summers , A. A. Taha , A. Tiulpin , S. A. Tsaftaris , B. Van Calster , G. Varoquaux , P. F. Jager , Nat. Methods 2024, 21, 195. 38347141 10.1038/s41592-023-02151-z PMC11182665

[49] C. Agarwal , O. Queen , H. Lakkaraju , M. Zitnik , Sci. Data 2023, 10, 144. 36934095 10.1038/s41597-023-01974-x PMC10024712

[50] H. Yuan , H. Yu , S. Gui , S. Ji , IEEE Trans. Pattern Anal. Mach. Intell. 2023, 45, 5782. 36063508 10.1109/TPAMI.2022.3204236

[51] K. Xu , W. Hu , J. Leskovec , S. Jegelka How Powerful are Graph Neural Networks?, arXiv:1810.00826 2018. 10.48550/arXiv.1810.00826

[52] L. Panconi , D. M. Owen , J. Griffie , Front Bioinf. 2023, 3, 1237551. 10.3389/fbinf.2023.1237551 PMC10704244 38076028

[53] M. Liu , Y. Luo , L. Wang , Y. Xie , H. Yuan , S. Gui , H. Yu , Z. Xu , J. Zhang , Y. Liu , K. Yan , H. Liu , C. Fu , B. M. Oztekin , X. Zhang , S. Ji , J. Mach. Learn. Res. 2021, 22, 10873.

