# Supplementary material for: ClusterNet: Classifying Single‐Molecule Localization Microscopy Datasets with Graph‐Based Deep Learning of Supracluster Structure
Source: Small Sci. 2025 Oct 9;5(12):e202500255. doi: 10.1002/smsc.202500255 (PMC12697834; doi:10.1002/smsc.202500255)
Supplement: Supplementary file 1 — Supplementary Material [file SMSC-5-e202500255-s001.pdf]

Supporting Information

ClusterNet: Classifying Single-Molecule Localization Microscopy Datasets with Graph-Based Deep Learning of Supra-Cluster Structure

Oliver Umney, Hayley Slaney, Christopher J. M. Williams, Philip Quirke, Michelle Peckham, Alistair P. Curd**

Text

Cell classification without filtering

To determine whether the preprocessing steps applied to the dataset impacted the classification, the dataset was generated again without filtering of the localizations during preprocessing or removing of cells based on the number of localizations. This gave 173 cells (no-response: 121, any-response: 52) from the same 23 patients. Classifying this dataset using the *ClusterNet-HCF* model with $k = 96$ clusters gave similar results to the dataset that included filtering of localizations and cells (AUROC: 0.72 ± 0.14, balanced accuracy: 0.63 ± 0.09). Despite not having an impact on this dataset, preprocessing steps may impact the performance on other SMLM datasets. Also, comparing performance with or without filtering may help to reveal the number of localizations and the precision required to successfully classify the structures.

Methods

LocNet architecture

PointTransformer v1 was adapted from the example in PyTorch Geometric. [44,46] The PointTransformer was composed of an initial multilayer perceptron (MLP), a transformer block, two transformer-down blocks, global maximum pooling from localizations into the cluster and a final output MLP (Figure S6).

First, the features for each localization node, i, were inputted to a MLP with ReLU activation function and batch normalisation, but as there were no input features for the localizations each feature was set to a dummy value (a vector of ones).

Next, the output localization node features from the MLP were inputted to a transformer block. Each transformer block was composed of an initial linear layer with ReLU activation, a PointTransformer convolution and a final linear layer with ReLU activation. For each localization node, i, the PointTransformer convolution, gave an output feature vector

$$\mathbf{x}'_i = \max_{j \in \mathcal{N}(i) \cup \{i\}} \alpha_{i,j}\left(\mathbf{W}_3\mathbf{x}_j + \delta_{ij}\right) \tag{1}$$

, where $\mathcal{N}(i)$ denoted the $\mathcal{N}$ nearest nodes, the vector attention coefficients were given by

$$\alpha_{i,j} = \mathrm{softmax}\left(\gamma_{\mathbf{\Theta}}\left(\mathbf{W}_1\mathbf{x}_i - \mathbf{W}_2\mathbf{x}_j + \delta_{i,j}\right)\right) \tag{2}$$

and the position embedding by

$$\delta_{i,j} = h_{\boldsymbol{\omega}}\left(\mathbf{p}_i - \mathbf{p}_j\right), \tag{3}$$

where $\mathbf{x}_i$ and $\mathbf{x}_j$ were the input features of nodes i and j, $\mathbf{p}_i$ and $\mathbf{p}_j$ were the 2D coordinates of nodes i and j, $\gamma_{\mathbf{\Theta}}$ and $h_{\boldsymbol{\omega}}$ were MLPs parameterised by $\mathbf{\Theta}$ and $\boldsymbol{\omega}$ respectively with batch normalization and ReLU activation function, and $\mathbf{W}_1, \mathbf{W}_2$ and $\mathbf{W}_3$ were learned weights of a linear layer. Here $\mathcal{N}$ was set to 5. The PointTransformer convolution calculated the maximum rather than the sum of neighboring features as was used in the original architecture. [46] This was because SMLM data is prone to artefacts, and maximum pooling can be robust to outliers and missing points and encourage the model to learn the overall structure. [45,51,52]

The output localization node features were then inputted to the transformer-down blocks. Each transformer-down block consisted of a transition down block, followed by a transformer block. In the transition down block, the localization node features were inputted to a MLP with ReLU activation function and instance normalization, then half of the nodes in the cluster were chosen and assigned the maximum of the features of their 5 nearest neighbors and themselves.

Finally, global maximum pooling aggregated the localization node features into the feature vector for the cluster, which was inputted to a MLP with a ReLU activation function, a plain last layer and no normalization giving the final embedding for the cluster.

Global maximum pooling was used rather than global mean pooling as in the original architecture, to be more robust to noise and outliers as outlined above. [46]

ClusterNet architecture

ClusterNet was composed of four PointTransformer message passing layers, a global maximum pooling layer and then a final linear layer. First, cluster node features were inputted to the four message passing layers. At each layer, the feature for each node was updated according to the PointTransformer convolution defined above, where the neighbors, $\mathcal{N}(i)$, of each cluster node, i, were defined by the edges from graph construction. After the fourth message passing layer, the cluster features were aggregated into a feature vector for the graph using maximum pooling. This was inputted to a linear layer followed by the log softmax function to give the log probabilities of the graph belonging to each class.

SubgraphX

We chose the 'split' method of assessing subgraphs, where nodes outside of a subgraph being assessed are removed from the graph. [21,53] This avoids the positions of the cluster nodes being set to (0, 0) coordinates in the 'zero_filling' method, which would have a greater effect on the supra-cluster structure and message passing in *ClusterNet*.
Further parameter values to those in Materials and Methods are in the table below. In our visualizations, self-loops and edge direction are not shown.

**Parameter**	**Value**	**Default value?**
rollout	100	No
min_atoms	5	yes
c_puct	10.0	yes
expand_atoms	14	yes
high2low	False	yes
local_radius	4	yes
sample_num	100	yes
reward_method	mc_l_shapley	yes
subgraph_building_method	split	No

Software used

The full list of Python dependencies can be found at https://github.com/oubino/locpix_points, however the following packages and software are highlighted below.

**Package(s)/Software**	**Used for**
Polars/PyArrow	Store localization data in Parquet files
PyTorch/PyTorch geometric	Represent data as graphs and create, train, and evaluate neural networks
Dask	Quantitative analysis
Numpy	Quantitative analysis
Open3D	Visualisation of point clouds and graphs
NetworkX	Generate high quality graph figures
Weights & Biases	Monitor network training and performance
Bokeh	Interactive visualisation of UMAPs
Dive into Graphs/PyTorch geometric	Graph explainability algorithms
Matplotlib/Seaborn/Adobe illustrator	Table and figure generation

Figures

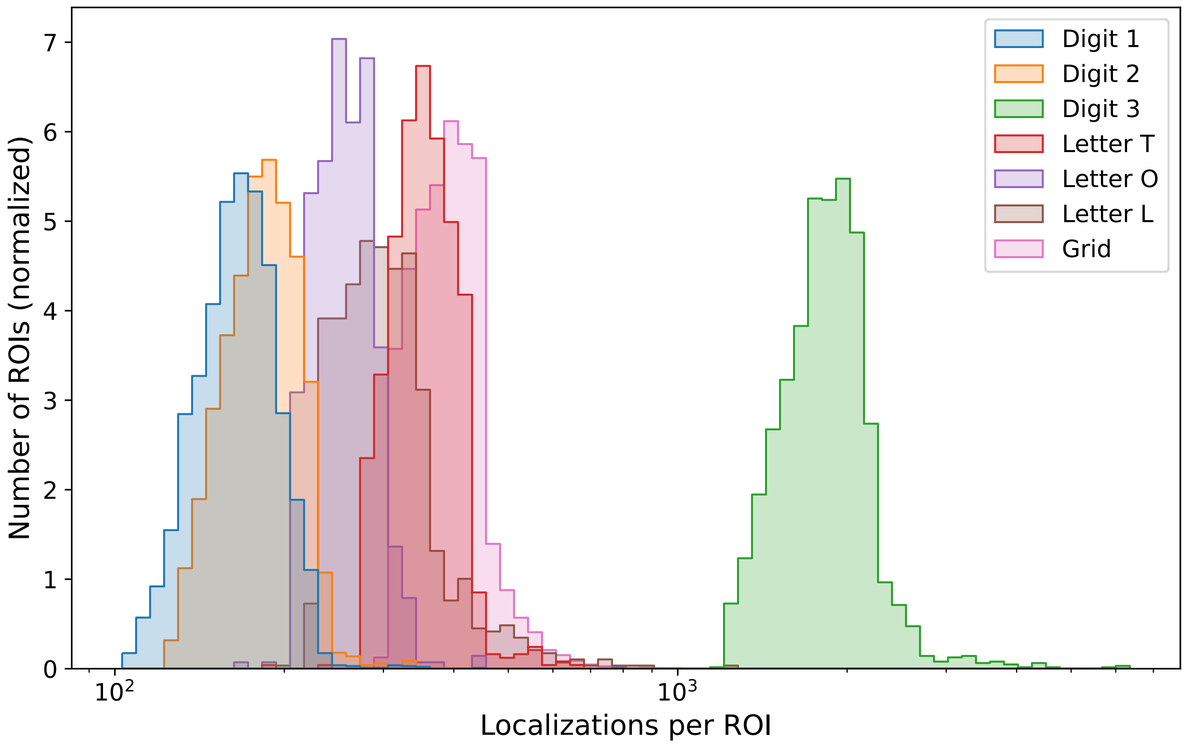

Figure S1. Histograms for the number of localizations per ROI for the different classes. Y-axis is normalized such that each histogram has the same area. X-axis is a log scale.

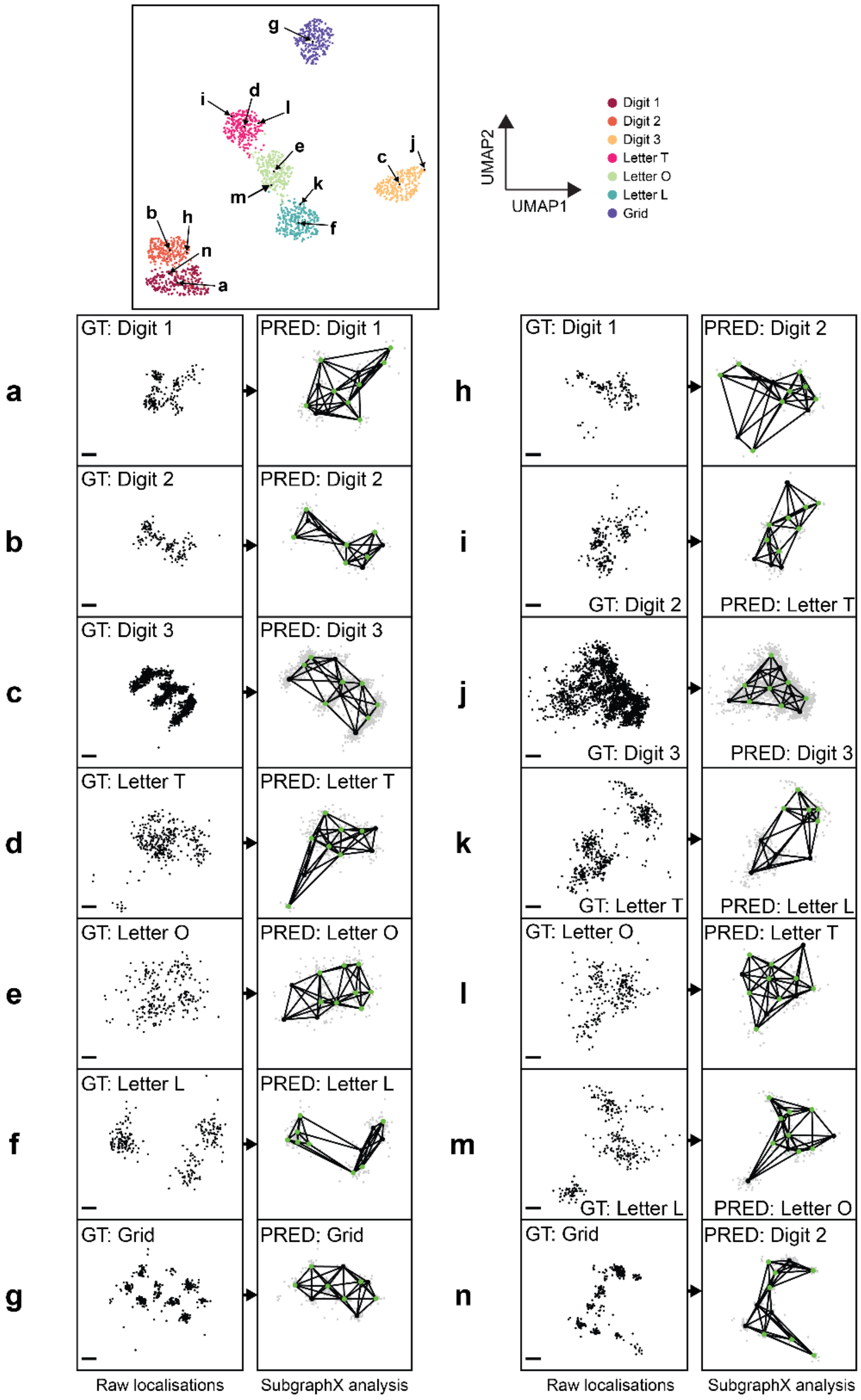

Figure S2. Structure analysis for *ClusterNet-HCF*. For each class, the centroid of the graph features from the 2D UMAP representation for the reserved test set is calculated (Top). The graph for each ground truth (GT) class with features closest to (**a-g**) or furthest from (**h-n**) their class centroid is identified (arrows in 2D UMAP). (**a-n**) SubgraphX analysis: the important subgraph is identified (green nodes) and the remaining cluster nodes (black dots), edges between clusters and localization nodes (grey dots) are also shown. PRED: Predicted class. Fidelity scores, ground truth and predictions by the model for graphs (**a-n**) are given in Table S7. Scale bar: 13 nm (Raw localizations).

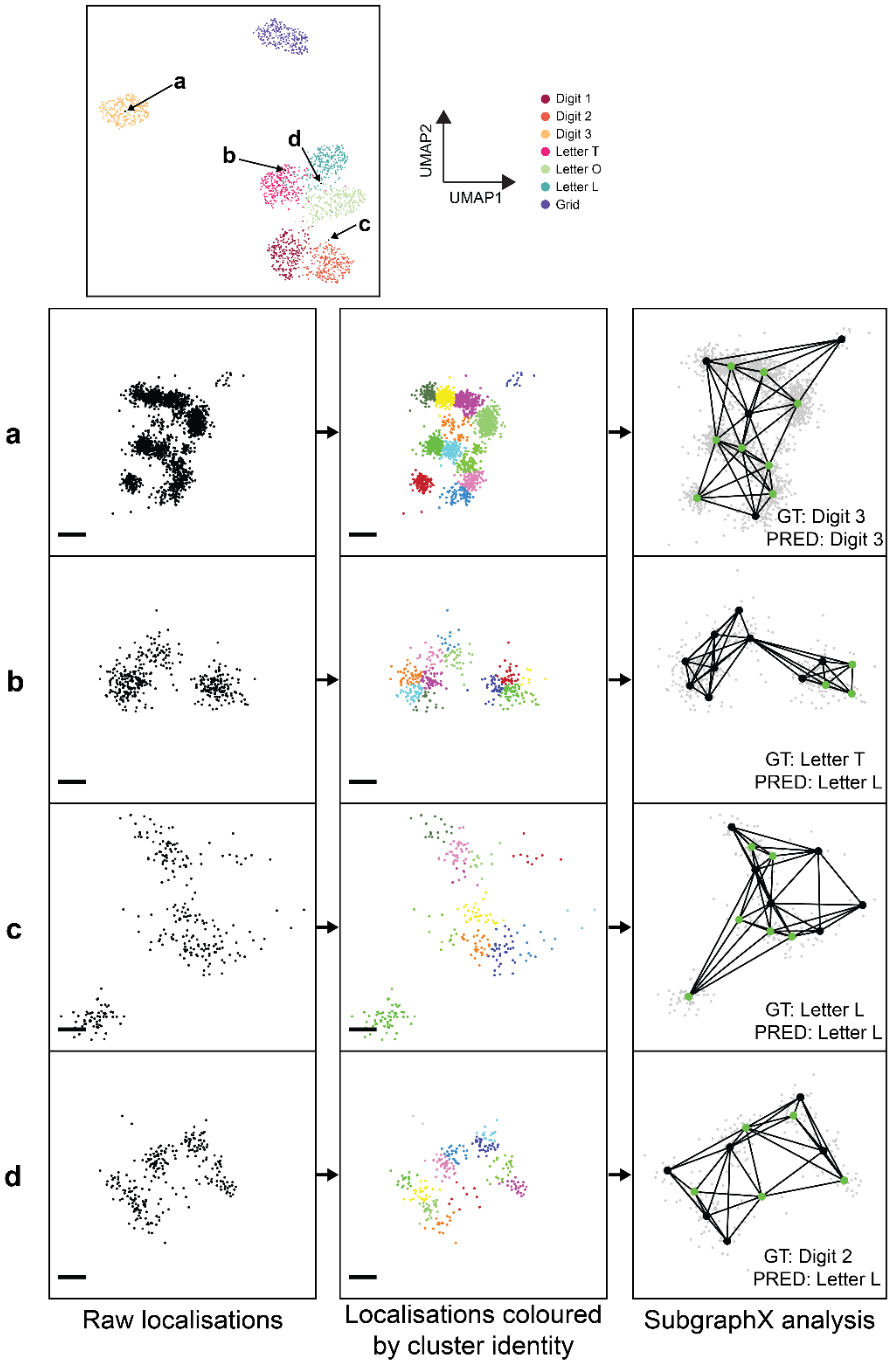

Figure S3. Structure analysis for *ClusterNet-LCF*. Four graphs (**a-d**) from the reserved test set are analyzed. (Top) 2D representation of the whole-graph deep features for the reserved test generated by UMAP, with the location of the graphs (**a-d**) indicated. SubgraphX analysis: the important subgraph is identified (green nodes) and the remaining cluster nodes (black dots), edges between clusters, localization nodes (grey dots), ground truth (GT) and prediction by the model (PRED) are also shown. Fidelity scores for graphs (**a-d**) are given in Table S8. Scale bar: 13 nm (raw localizations and localizations colored by cluster identity).

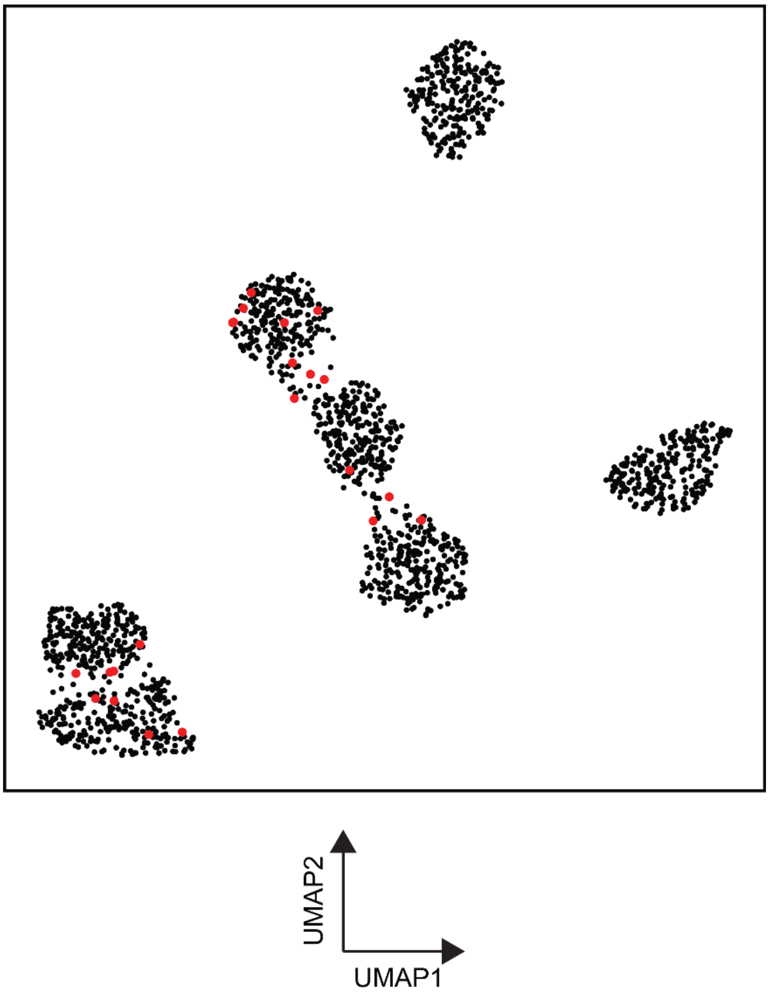

Figure S4. Feature analysis for incorrectly classified graphs. 2D representation of the whole-graph features colored according to whether they were correctly (black) or incorrectly (red, larger for clarity) classified (*ClusterNet-HCF*, reserved test dataset).

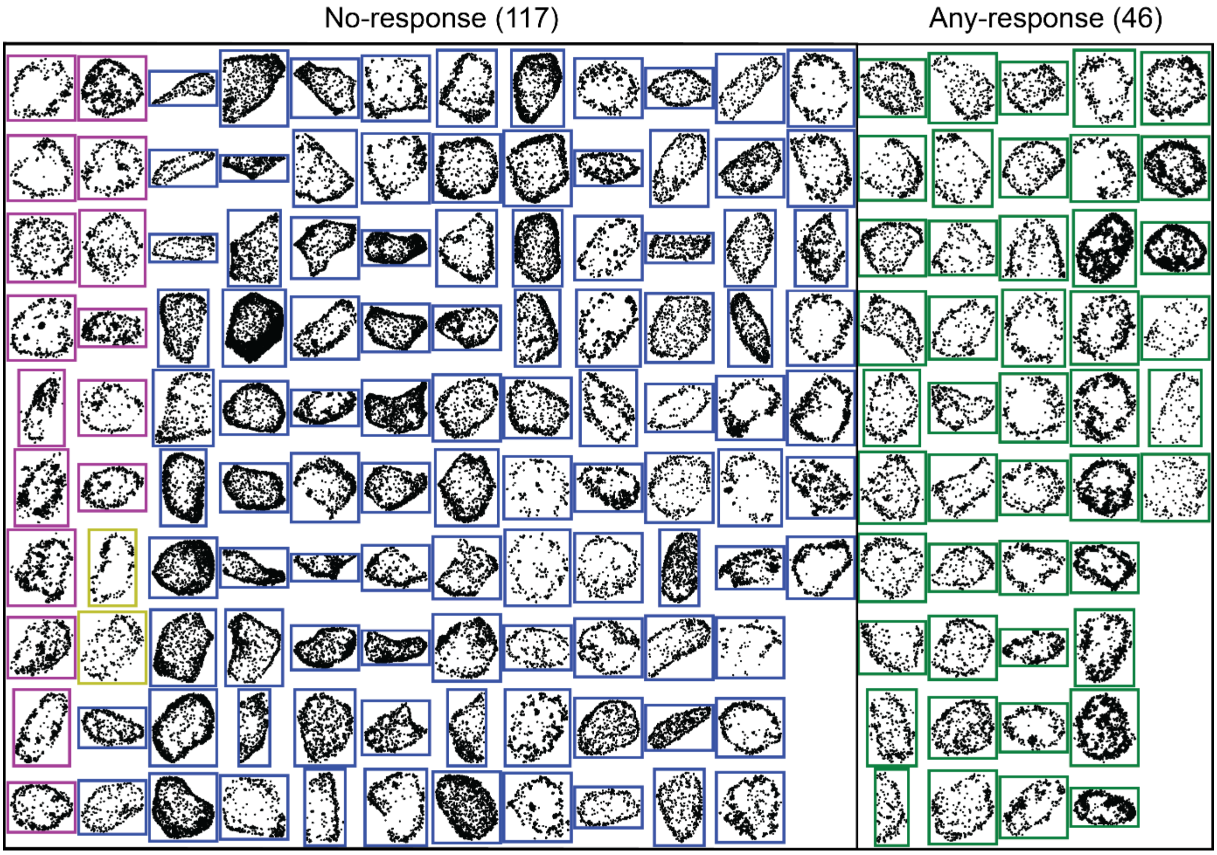

Figure S5. EREG localizations for each cell in the tumor dataset. Borders colored by response to anti-EGFR treatment: death (pink), clinical progression (yellow), radiological progression (blue) and partial response (green). Each cell is rescaled independently to approximately the same size for the plot, therefore, cell sizes should not be compared. Number in brackets indicates the number of cells for that class.

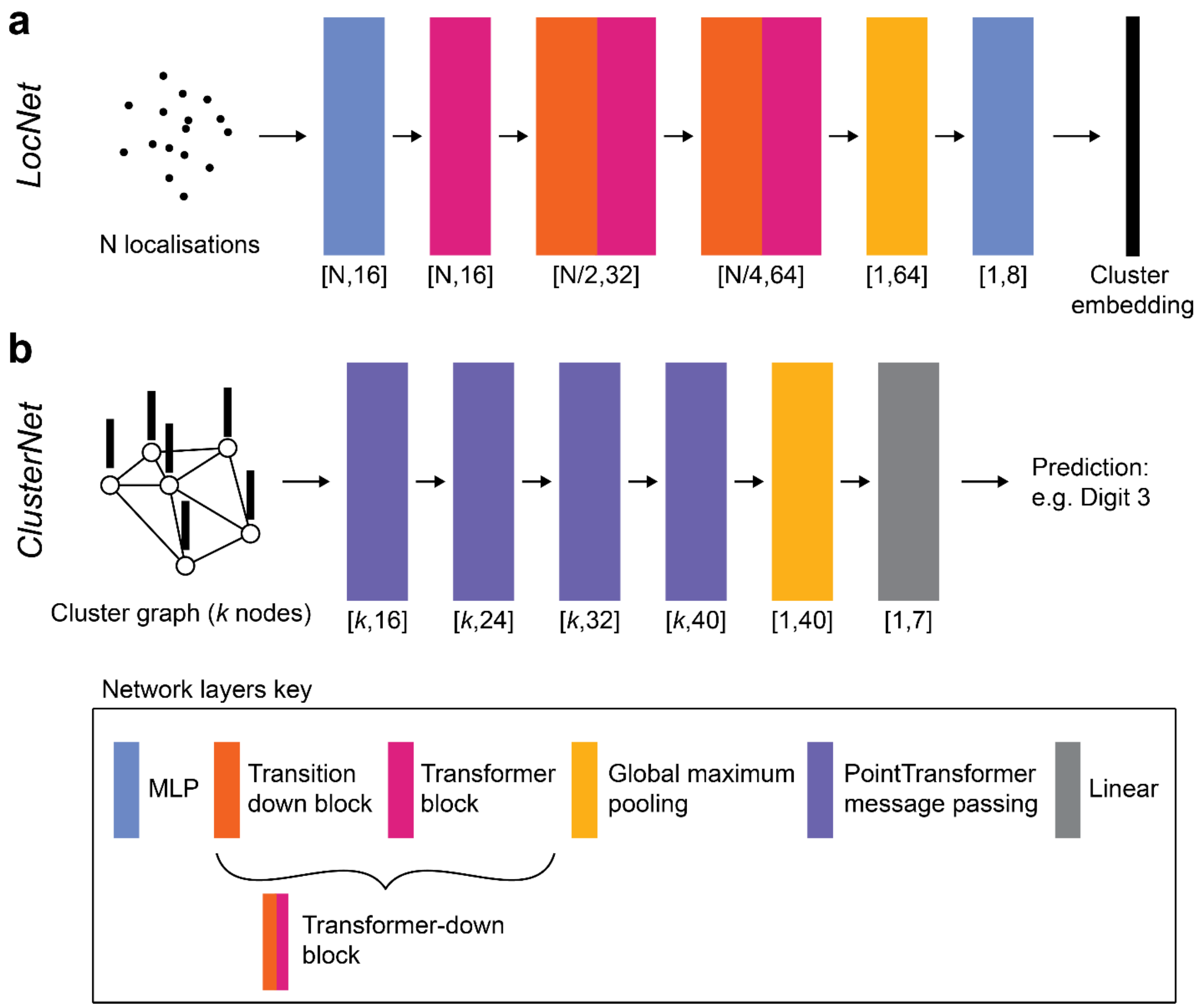

Figure S6. *LocNet* and *ClusterNet* architectures. **a** *LocNet* transforms input SMLM localizations for a cluster into a cluster embedding, via a PointTransformer v1 based network.[46] **b** *ClusterNet* classifies cluster graph, *k* nodes with handcrafted or *LocNet* embedded features, using PointTransformer based convolutions as message passing layers. Output dimensions of each layer are given in square brackets [X,Y], where X: number of localizations (**a**) or nodes (**b**), Y: per localization/node feature size.

Tables

Table S1. Characterizing the DNA-PAINT digits and letters dataset. σ: localization uncertainty.

Class	Min. localizations per ROI	Max. localizations per ROI	Mean localizations per ROI	Min. σ [nm]	Max. σ [nm]	Mean σ [nm]
Digit 1 (n=4155)	103	788	167	9	30	16
Digit 2 (n = 4943)	127	645	184	9	94	15
Digit 3 (n = 2541)	1186	6360	1855	9	37	15
Letter T (n = 991)	185	650	357	11	38	21
Letter O (n = 560)	167	454	258	13	38	24
Letter L (n = 1161)	197	1228	313	6	31	13
Grid (n = 7696)	305	1244	395	8	73	13

Table S2. Performance of *ClusterNet-HCF* and *ClusterNet-LCF* on the training folds from k-fold training. Results for each class are averaged over the training folds. AUROC = area under the receiver operator curve.

Model	Metric	Digit 1	Digit 2	Digit 3	Letter T	Letter O	Letter L	Grid	Mean ± S.D.
ClusterNet-HCF	Recall	0.98	0.96	1.00	0.99	1.00	1.00	0.99	0.99 ± 0.01
ClusterNet-LCF		0.97	0.97	1.00	0.97	0.98	0.95	1.00	0.98 ± 0.02
ClusterNet-HCF	AUROC	1.00	1.00	1.00	1.00	1.00	1.00	1.00	1.00 ± 0.00
ClusterNet-LCF		1.00	1.00	1.00	1.00	1.00	1.00	1.00	1.00 ± 0.00

Table S3. Performance of *ClusterNet-HCF* and *ClusterNet-LCF* on the validation folds from k-fold training. Results for each class are averaged over the validation folds. AUROC = area under the receiver operator curve.

Model	Metric	Digit 1	Digit 2	Digit 3	Letter T	Letter O	Letter L	Grid	Mean ± S.D.
ClusterNet-HCF	Recall	0.98	0.97	1.00	0.97	0.95	0.99	0.99	0.98 ± 0.02
ClusterNet-LCF		0.96	0.97	1.00	0.93	0.88	0.92	1.00	0.95 ± 0.04
ClusterNet-HCF	AUROC	1.00	1.00	1.00	1.00	1.00	1.00	1.00	1.00 ± 0.00
ClusterNet-LCF		1.00	1.00	1.00	1.00	1.00	1.00	1.00	1.00 ± 0.00

Table S4. Performance of *ClusterNet-HCF* and *ClusterNet-LCF* on the test folds from k-fold training. Results for each class are averaged over the test folds. AUROC = area under the receiver operator curve.

Model	Metric	Digit 1	Digit 2	Digit 3	Letter T	Letter O	Letter L	Grid	Mean ± S.D.
ClusterNet-HCF	Recall	0.98	0.96	1.00	0.97	0.94	0.99	0.99	0.98 ± 0.02
ClusterNet-LCF		0.96	0.96	1.00	0.92	0.90	0.91	0.99	0.95 ± 0.04
ClusterNet-HCF	AUROC	1.00	1.00	1.00	1.00	1.00	1.00	1.00	1.00 ± 0.00
ClusterNet-LCF		1.00	1.00	1.00	1.00	1.00	1.00	1.00	1.00 ± 0.00

Table S5. Confusion matrix for *ClusterNet-LCF* on the reserved test set.

		**Predicted**						
		Digit 1	Digit 2	Digit 3	Letter T	Letter O	Letter L	Grid
**Actual**	Digit 1	233	4	0	1	2	0	0
	Digit 2	11	226	0	0	2	1	0
	Digit 3	0	0	239	0	0	0	1
	Letter T	1	0	0	224	5	10	0
	Letter O	2	0	0	5	227	6	0
	Letter L	0	0	0	14	7	219	0
	Grid	0	1	0	0	0	2	237

Table S6. Confusion matrix for *ClusterNet-HCF* on the reserved test set.

		**Predicted**						
		Digit 1	Digit 2	Digit 3	Letter T	Letter O	Letter L	Grid
**Actual**	Digit 1	238	2	0	0	0	0	0
	Digit 2	5	234	0	1	0	0	0
	Digit 3	0	0	240	0	0	0	0
	Letter T	3	1	0	231	3	1	1
	Letter O	0	0	0	1	238	1	0
	Letter L	0	0	0	0	2	238	0
	Grid	0	1	0	0	0	0	239

Table S7. Extended classification and structure analysis results for *ClusterNet-HCF*. Results for the graphs in Figure S2a-n. These include positive and negative fidelity scores for subgraphs identified by SubgraphX. Positive fidelity ranges from 1 (best performance) to 0 (worst performance). Negative fidelity ranges from 0 (best performance) to 1 (worst performance).

Graph	Ground truth	Prediction	Positive fidelity	Negative fidelity
a	Digit 1	Digit 1	1.0	4.0×10^{-4}
b	Digit 2	Digit 2	1.0	4.0×10^{-4}
c	Digit 3	Digit 3	1.0	2.6×10^{-5}
d	Letter T	Letter T	0.99	0.04
e	Letter O	Letter O	1.0	1.0×10^{-7}
f	Letter L	Letter L	6.0×10^{-7}	3.1×10^{-6}
g	Grid	Grid	1.0	1.0×10^{-4}
h	Digit 1	Digit 2	0.70	0.30
i	Digit 2	Letter T	0.62	0.16
j	Digit 3	Digit 3	1.0	3×10^{-4}
k	Letter T	Letter L	0.99	8.9×10^{-3}
l	Letter O	Letter T	0.96	2.5×10^{-2}
m	Letter L	Letter O	0.81	0.20
n	Grid	Digit 2	0.45	0.40

Table S8. Extended structure analysis results for *ClusterNet-LCF*. Positive and negative fidelity scores for subgraphs identified by SubgraphX for the graphs (**a-d**) in Figure S3. Positive fidelity ranges from 1 (best performance) to 0 (worst performance). Negative fidelity ranges from 0 (best performance) to 1 (worst performance).

Figure	Positive fidelity	Negative fidelity
a	1.00	1.34×10^{-5}
b	0.56	0.30
c	0.88	0.12
d	0.79	0.14

Table S9. Characterizing the cell dataset with or without filtering by localization parameters and counts per cell. σ: localization uncertainty (mean of uncertainty in x and y).

Class	Min. Localizations per cell	Max. localizations per cell	Mean localizations per cell	Min. σ [nm]	Max. σ [nm]	Mean σ [nm]
No-response unfiltered (n = 121)	2,211	80,753	13,197	0.1	275.7	25.7
Any-response unfiltered (n = 52)	1,933	40,439	8,451	0.2	304.5	24.6
No-response filtered (n = 117)	508	26,767	3,820	0.6	25.0	9.8
Any-response filtered (n = 46)	516	15,304	2,670	0.7	24.9	10.3

Table S10. Classification performance of *ClusterNet-HCF* on the cells ($n_{\text{no-response}} = 117$, $n_{\text{any-response}} = 46$) from advanced colorectal cancer patients ($n_{\text{no-response}} = 18$, $n_{\text{any-response}} = 5$). Performance metric scores are presented as the mean ± standard deviation over the five test folds.

Clustering algorithm	AUROC	Balanced accuracy
k-means (*k* = 12)	0.60 ± 0.13	0.55 ± 0.12
k-means (*k* = 24)	0.51 ± 0.08	0.40 ± 0.08
k-means (*k* = 48)	0.65 ± 0.20	0.57 ± 0.12
k-means (*k* = 72)	0.66 ± 0.17	0.61 ± 0.12
k-means (*k* = 96)	**0.70 ± 0.15**	**0.64 ± 0.14**
k-means (*k* = 120)	0.66 ± 0.18	0.58 ± 0.14
k-means (*k* = 144)	0.68 ± 0.21	0.63 ± 0.16
DBSCAN (ε = 50 nm, minPts = 3)	0.70 ± 0.20	0.63 ± 0.19
DBSCAN (ε = 50 nm, minPts = 5)	0.44 ± 0.25	0.51 ± 0.14
DBSCAN (ε = 50 nm, minPts = 7)	0.35 ± 0.25	0.42 ± 0.19
DBSCAN (ε = 75 nm, minPts = 3)	0.65 ± 0.17	0.65 ± 0.18
DBSCAN (ε = 75 nm, minPts = 5)	0.45 ± 0.27	0.49 ± 0.13
DBSCAN (ε = 75 nm, minPts = 7)	0.41 ± 0.32	0.52 ± 0.18
DBSCAN (ε = 100 nm, minPts = 3)	0.60 ± 0.21	0.59 ± 0.14
DBSCAN (ε = 100 nm, minPts = 5)	0.63 ± 0.13	0.60 ± 0.11
DBSCAN (ε = 100 nm, minPts = 7)	0.48 ± 0.32	0.52 ± 0.17
